# Loss to follow-up in a randomized controlled trial study for pediatric weight management (EPOC)

**DOI:** 10.1186/s12887-016-0727-2

**Published:** 2016-11-14

**Authors:** Petra Warschburger, Katja Kröller

**Affiliations:** 1 Department of Psychology, University of Potsdam, Counseling Psychology, Potsdam University, Karl- Liebknecht- Str. 24/25, 14476 Potsdam, Germany; 2 Department of Health Psychology, H:G Hochschule für Gesundheit & Sport, Technik & Kunst, Vulkanstraße 1, 10367 Berlin, Germany

**Keywords:** Attrition, Obesity, Child, Predictors, Weight management trial

## Abstract

**Background:**

Attrition is a serious problem in intervention studies. The current study analyzed the attrition rate during follow-up in a randomized controlled pediatric weight management program (EPOC study) within a tertiary care setting.

**Methods:**

Five hundred twenty-three parents and their 7–13-year-old children with obesity participated in the randomized controlled intervention trial. Follow-up data were assessed 6 and 12 months after the end of treatment. Attrition was defined as providing no objective weight data. Demographic and psychological baseline characteristics were used to predict attrition at 6- and 12-month follow-up using multivariate logistic regression analyses.

**Results:**

Objective weight data were available for 49.6 (67.0) % of the children 6 (12) months after the end of treatment. Completers and non-completers at the 6- and 12-month follow-up differed in the amount of weight loss during their inpatient stay, their initial BMI-SDS, educational level of the parents, and child’s quality of life and well-being. Additionally, completers supported their child more than non-completers, and at the 12-month follow-up, families with a more structured eating environment were less likely to drop out. On a multivariate level, only educational background and structure of the eating environment remained significant.

**Conclusions:**

The minor differences between the completers and the non-completers suggest that our retention strategies were successful. Further research should focus on prevention of attrition in families with a lower educational background.

**Trial registration:**

Current Controlled Trials ISRCTN24655766. Registered 06 September 2008, updated 16 May 2012.

## Background

Attrition is a serious problem in intervention studies, which can threaten reliability and validity for several reasons: First, when participants drop out of a study, analyses are limited to a smaller sample, which can lead to insufficient statistical power. Second, although participants may drop out for various reasons, some of these can be related to the outcome. Third, the drop-out pattern may differ between treatment groups, altering the random composition of the groups and leading to confounding influences. Therefore, high attrition rates can cause a significant bias in the study. Such a bias might alter the characteristics of a sample and impede the comparability to the original sample. Moreover, the bias might alter the covariance of the variables included, possibly affecting outcomes or other correlated variables (as reviewed by Ahern [1]).

Reviewing the attrition rates in randomized controlled studies of chronic illness in childhood, Karlson and Rapoff [2] reported that on average, 37 % (range 0–75 %) refused to take part in the study at the beginning, 4 % (range 0–35 %) provided no baseline data, 20 % (range 0–54 %) no initial follow-up data, and 32 % (range 0–59 %) no long-term follow-up data. The majority of the studies did not analyze differences between completers and non-completers. Collins et al. [3] assessed the efficacy of dietetic interventions for children with obesity. From the 37 randomized controlled trials (RCT) they extracted, 26 included follow-up data (1 month to 10 years). Attrition rates during follow-up varied from 5 to 37 %. Only 9 studies reported efficacy data on the basis of an intention-to-treat analysis.

In general, only a small number of studies have reported explicit loss at follow-up. In a recent family-focused intervention study [4], approximately 20 % of participants dropped out at the follow-up assessments. These children were significantly older and had a higher body mass index (BMI) z-score at baseline. This is in line with the findings of van den Aker et al. [5]_,_ who reported an attrition rate of 33 % at the 1-year follow-up, with drop-outs being older and less successful during treatment. McCallum et al. [6] stated that the children lost in follow-up were slightly heavier, and that their parents reported a lower quality of life for their children. Unfortunately, it was not reported whether these differences were statistically significant. Hughes et al. [7] observed a 34.9–36.9 % loss during follow-up 12 months after the start of treatment, and did not report any demographic differences between completers and non-completers. This is in line with the data of Savoye et al. [8], who found no differences between completers and non-completers with respect to baseline characteristics (BMI, age, gender).

Other studies examined the determining factors for drop-out during treatment [9], with fairly inconclusive results, as almost no overlap was found between the indicators analyzed. Although some studies reported a higher attrition rate for those with a higher BMI z-score [10–13], others did not find a significant effect (n.s. [14, 15]), older age [10, 11, 13] (n.s. [14–16]), ethnic minority status [10, 14, 15] (n.s. [11, 16]), lower socioeconomic status (SES)/education (trend [17]; n.s. [12, 16]) or psychological problems [10, 13, 17] (n.s. [11, 12]). Consistently, no study reported gender effects [11–13, 15–17].

To be able to adequately interpret study results, it is crucial to achieve high retention rates in intervention research. Participants’ motivation to devote time and effort to filling in questionnaires or attending medical check-ups will decrease over time, and this may be particularly true when treatment takes place in inpatient facilities that are far from patients’ homes. Hence, knowledge regarding predictors of attrition during follow-up in clinical trials is limited. Therefore, we analyzed the data of a large randomized controlled study (Empowering Parents of Obese Children – EPOC study) in order to examine which factors predicted failure to provide objective weight-related measurements. We hypothesized that parents with a lower educational background and children experiencing less success during treatment and higher psycho-social strain would return objective weight data less frequently.

## Methods

### Sample and procedure

Written consent was obtained from parents of 7–13-year-old children with obesity to take part in the EPOC study examining the effects of supplementary parent training as part of the child’s inpatient obesity intervention. Inclusion and exclusion criteria are described in detail elsewhere [18]. The study was approved by the Ethics Committee of the University of Potsdam on 19 May 2006.

Children’s weight and height were measured by a physician, who was blind to the treatment group to which they were assigned, at different time periods: at baseline (beginning of the intervention), post-intervention and six and twelve months thereafter. In addition, parents and their children filled in a set of questionnaires. Table 1 provides a sample description of the study’s 523 children (274, 52.4 % girls) and their parents at baseline.

**Table 1:** Sample description of the total sample at baseline

	Children (*n* = 523)
Sex	
female	52.4 %
male	47.6 %
Age (in years of age)	11.3 ± 1.29 (7–13)
BMI- SDS (measured)	2.56 ± 0.39 (1.89–4.57)
Weight classification	
obese^a^	57.2 %
extremely obese^b^	42.8 %
	Parents (*n* = 523)
Age (in years of age)	40.56 ± 5.77 (25.94–58.78)
Educational level (in years of school completed)	10.3 ± 1.40 (6–13)
Family status	
relationship	68.8 %
single	31.2 %
BMI (self-reported in kg/m^2^)	29.19 ± 6.89 (14.10–59.06)
Weight classification	
normal- or under-weight^c^	32.2 %
overweight^d^	28.7 %
obese^e^	39.1 %

*Note: BMI-SDS* body mass index - standard deviation score

^a^ 97th percentile < BMI-SDS ≤ 99.5th percentile based on the classification by Kromeyer-Hauschild et al. [19]

^b^ BMI-SDS > 99th percentile

^c^ BMI ≤ 25

^d^ 25 < BMI ≤ 30

^e^ BMI > 30

### Measurements

#### Demographic and weight data

Parents were asked to provide demographic information such as age, family status and educational level (number of years spent in school) at the beginning of their child’s inpatient stay. In addition, all parents reported their own height and weight, as well as the respective data of their partners. Children were weighed in light underwear by a physician on a standard beam scale (accurate to 100 g) and measured using a calibrated stadiometer (accurate to 1 cm). Thereafter, a standardized BMI (BMI-SDS) for age and sex was calculated [19]. Several steps were taken to minimize loss to follow-up. To obtain objective weight data, we conducted a 6- and 12-month follow-up by contacting families and physicians by post. If families did not provide the weight data within 3 weeks, a reminder was sent by post. For the 12-month follow-up, we also telephoned the families that had still not replied and encouraged them to visit their physician or a pharmacy in order to provide the data. As a final option, we offered them a home visit. Both families and physicians were reimbursed for returning the respective questionnaire (50 and 25€, respectively).

#### Child factors

Since the results regarding the factors that influence the attrition rate in clinical trials are inconsistent, we analyzed a wide range of child and parental factors. All data with the exception of the child’s self-efficacy are based on parent reports. The health-related quality of life (HRQoL) was measured with the KID-KINDL-R [20]. The total score (including the 4 subscales ‘psychological well-being’, ‘self-esteem’, ‘family’ and ‘peers’) revealed an acceptable reliability value (α = .85) comparable with the original form (α = .70 [21]). The child’s weight-related quality of life (WRQoL) was measured with an established German instrument [22]. In line with previous validation values (α = .87 [23]), the reliability was good (α = .85). In addition, parents reported on the child’s food intake pertaining to ‘problematic’ (e.g., sweets, salty snacks, fast food and soft drinks) and ‘healthy’ (fruits, vegetables) food items on a 5-point scale (“never” – “several times a day”). Latent structure models confirmed the postulated factor structure [24]. The child’s eating behavior was measured with the FKE-KJ [22], which combines the 2 subscales ‘speed of eating’ (4 items) and ‘family eating environment’ (4 items). In accordance with previous evaluations, both scales showed an acceptable reliability within the current sample (α = .77 and .70, respectively). Furthermore, the child’s activity level, including media consumption and physical exercise, was assessed with an instrument from the KIGGS-study [25]. The instrument includes the mean duration (in hours) of the child’s use of television, video or computers as well as physical activity assessed by a 5-point Likert-scale. We formed a summarized score indicating the overall media consumption and activity level during an entire week. The child’s psychosocial strain was measured with the Strengths and Difficulties Questionnaire SDQ [26]. The sum score is made up of four subscales: ‘emotional problems’, ‘behavioral problems’, ‘peer problems’ and ‘hyperactivity’. The scale showed good reliability (α = .84). Children assessed their own weight-related self-efficacy, answering the GW-SW-KJ [22], which includes 24 items rated on a 5-point Likert scale. Previous reliability values (α = .89) were replicated in the current study (α = .89). All scales were transformed to a 0–100 scale.

#### Parental aspects

Parental quality of life was measured by the well-established SF12 [27], including physical and mental well-being. The parents’ perception of their support of their child was measured with a self-constructed instrument including aspects such as being a role model, and emotional and instrumental support. The postulated factor structure was confirmed [24], showing acceptable reliability values for the summarized scale (α = .80). The parental self-efficacy in changing family eating and activity habits even in the face of problems or challenges was measured by a further self-constructed scale that includes aspects of care-taking, personal changes made and social demands. The Cronbach’s α indicated a high reliability of the scale (α = .95).

### Statistical analyses

All statistical analyses were performed using SPSS 21.0. Attrition was defined as missing objective weight data at the 6- or 12-month follow-up. In the first step, we analyzed univariate differences between completers and non-completers (Chi-square, (M)ANOVA) and in the second step, we used logistic regression analysis to explore the variables that were associated with a higher risk of quitting the study on a multivariate level. For the logistic regression, variables were included stepwise: (1) demographic and inpatient stay-related aspects, (2) parental aspects, (3) the child’s psychosocial strain, and (4) secondary treatment outcomes, such as the child’s quality of life and self-efficacy, as well as activity level, food intake and eating behavior.

## Results

At the end of the intervention, the data of one child could not be obtained. Therefore, only the data from 522 children were available. Complete weight and height data were available for 259 (49.6 %) children at the 6-month follow-up and for 350 (67.0 %) at the 12-month follow-up.

### Differences between completers and non-completers

Univariate differences between completers and non-completers at the 6-month follow-up are presented in Table 2. The children’s initial weight status and weight change during the inpatient stay differed significantly between completers and non-completers, indicating a higher initial weight status and a lower weight loss in non-completers.

**Table 2:** Comparison of completers and non-completers at 6-month follow-up

	Completers (*n* = 259)	Non-Completers (*n* = 264)	F (*X* ^2^)	p	η^2^
Children					
Sex					
female	135	139	.02	.90	
male	124	125			
Age (years)	11.24	11.34	.85	.36	<.001
Initial BMI-SDS	**2.52**	**2.60**	**5.63**	**.02**	**.01**
Duration of inpatient stay (days)	39.67	39.69	<0.001	.98	<.001
Study arm					
IG	130	119	1.37	.24	
CG	129	145			
Weight change during stay (BMI-SDS)	**−0.35**	**−0.32**	**6.77**	**.01**	**.01**
Health-related QoL^a^	67.94	66.28	2.48	.12	.01
Weight-related QoL^a^	46.40	44.08	2.24	.14	<.001
Self-efficacy^a^	55.91	57.97	2.26	.13	.01
Psychosocial strain	6,41	6,35	<.001	.96	<.001
Food intake					
healthy^a^	69.46	65.00	3.06	.08	.01
Problematic^a^	48.69	47.28	.87	.35	<.001
Eating					
speed^a^	35.67	34.68	.29	.59	<.001
Structure^a^	**72.46**	**69.66**	**4.19**	**.04**	**.01**
Media consumption^a^	12.06	11.74	.08	.78	<.001
Activity level^a^	1.06	1.05	.02	.90	<.001
Parents					
Age (years)	41.05	40.06	3.15	.08	.01
Educational level (years of school)	10.37	10.24	.95	.33	<.001
Family status					
in relationship	149	142	.14	.71	
single	65	67			
BMI (kg/m^2^; self-reported)	28.91	29.46	.68	.41	<.001
LQ					
mental^a^	48.71	47.71	1.18	.28	<.001
Physical^a^	50.05	49.65	.29	.59	<.001
Self-efficacy^a^	64.99	63.77	.85	.36	<.001
Child’s support^a^	73.07	71.66	2.28	.13	<.001

*Note: F* statistical test value, *p* significance value, *η2* explained variance

^a^ mean scores on a scale ranging from 0 to 100

These differences between completers and non-completers at the 6-month follow-up were also observed in the one-year follow-up (see Table 3). In addition, there were significant differences regarding the parents’ educational level (completers had a higher level of education than non-completers) and parental support (completers reported more support for their child than non-completers), as well as the child’s psychosocial strain (completers reported less psychosocial strain than non-completers), the child’s WRQoL (completers reported higher scores than non-completers), and family eating environment (completers reported higher scores than non-completers).

**Table 3:** Comparison of completers and non-completers at 12-month follow-up

	Completers (*n* = 350)	Non-Completers (*n* = 173)	F (*X* ^2^)	p	η^2^
Children					
Sex					
female	177	97	1.40	.24	
male	173	76			
Age (years)	11.28	11.34	0.28	.60	<.001
BMI-SDS (measured)	2.52	2.63	8.53	<.001	.02
Duration of inpatient stay (days)	39.95	39.14	0.76	.39	<.001
Study arm					
IG	169	80	0.19	.66	
CG	181	93			
Weight change during stay (BMI-SDS units)	−0.35	−0.32	5.04	.03	.01
Health-related QoL^a^	67.81	65.65	3.66	.06	.01
Weight-related QoL^a^	46.79	41.97	8.51	<.001	.02
Self-efficacy^a^	57.01	56.79	0.02	.88	<.001
Psychosocial strain^a^	12.89	14.42	5.85	.02	.01
Food intake					
healthy^a^	68.23	65.16	1.25	.26	<.001
Problematic^a^	47.76	48.52	0.22	.64	<.001
Eating					
speed^a^	35.52	34.45	0.29	.59	<.001
Structure^a^	72.42	68.18	8.34	<.001	.02
Media consumption^a^	23.83	24.34	0.19	.66	<.001
Activity level^a^	1.12	0.93	3.38	.07	.01
Parents					
Age	40.62	40.42	0.11	.74	<.001
Educational level (years of school)	10.41	10.08	4.77	.03	.01
Family status					
relationship	206	85	2.63	.11	
single	83	49			
BMI (kg/m^2^; self-reported)	29.21	29.15	0.01	.94	<.001
QoL					
mental^a^	48.45	47.72	0.55	.46	<.001
Physical^a^	49.83	49.89	0.01	.95	<.001
Self-efficacy^a^	64.32	64.54	0.02	.88	<.001
Child’s support^a^	73.09	70.83	5.12	.02	.01

*Note: F* statistical test value, *p* significance value, *η2* explained variance, *QoL* quality of life, *IG* intervention group, *CG* control group, *BMI-SDS* body mass index - standard deviation score

^a^ mean scores on a scale ranging from 0 to 100

### Drop-out rates 6 and 12 months after the intervention

The logistic regression to predict those families that missed the weight and height measurement explained 16 % of the variance after 6 months and 14 % after 12 months. It was also able to predict 60 % of the sample correctly after 6 months and 67 % after 12 months. At the 6-month follow-up, participation in the intervention group (odds ratio (OR)) = 0.60) and having older parents (OR = 0.95) was associated with a lower risk of dropping out, whereas a lower weight loss during the inpatient stay was associated with a higher risk (OR = 24.05). After 12 months, higher parental educational level (OR = 0.81) and a more structured family eating environment (OR = 0.97) were associated with a lower risk of dropping out. Table 4 shows the results for the 12-month follow-up.

**Table 4:** Logistic regression to predict drop-out at 12-month follow-up

	Wald	df	*p*	Exp(B)	95 % CI
Children					
Sex	1.715	1	.190	1.386	.850–2.260
Age (years)	0.070	1	.791	1.025	.856–1.227
BMI-SDS (measured)	3.278	1	.070	1.870	.950–3.682
Study arm	1.038	1	.308	.785	.492–1.251
Weight change during stay	0.396	1	.529	1.886	.261–13.621
Weight-related QoL	0.260	1	.610	0.996	.982–1.011
Psychosocial strain	1.216	1	.270	1.023	.983–1.064
Eating					
structure	8.429	1	.004	.977	.962–0.992
Parents					
Educational level (years of school)	4.038	1	.044	.839	.707–0.996
Child’s support	0.117	1	.733	.996	.974–1.019

*Note*: *Wald* statistical test, *df* degrees of freedom, *p* significance level, *Exp(B)* odds ratio, *95 % CI* confidence interval, *QoL* quality of life, *BMI-SDS* body mass index - standard deviation score

## Discussion

Attrition is a highly relevant and prevalent problem in pediatric weight management trials. In the current study, we observed an attrition rate of 33.0 % at the 12-month follow-up. Our results are in line with those reported in the literature [3, 5, 7]. A recent review reported higher attrition rates in studies that focused on children with overweight or obesity (79.6 %) as well in long-term studies (74 %) [28]. Based on the results of a previous study assessing parental factors which might impede or facilitate their participation [29],^8^ we assumed time and financial constraints to be important. Therefore, we used an expensive and time-consuming method (re-sending questionnaires and reminder postcards, repeated telephone calls, offering home visits and financial incentives). These strategies were considered as successful retention strategies to facilitate parents’ participation and receive the long-term data [30]. We assume that this strategy did at least pay off a little, since we invested the greatest efforts before the second follow-up and the attrition rate at 12 months was slightly lower than that at the 6-month follow-up. Nevertheless, the attrition rate was high, and retention rates fell below our expectations. Further studies are needed to indicate empirically validated retention strategies. In order to enhance study commitment and decrease attrition, the study by Germann et al. [31] used an orientation session to give information about achievable and healthy weight loss. We agree that this might be helpful in decreasing the number of unsuccessful weight loss attempts, but it also excludes many individuals with weight problems and focuses only on highly motivated patients. Further research is needed on how to increase the retention rate in clinical trials, especially when not only self-reports but also physiological measurements are required [28].

BMI at baseline and weight loss during intervention are often reported to influence the drop-out in obesity interventions. With respect to the baseline BMI, we observed a higher drop-out rate in families whose children were heavier at baseline [4, 6]. In line with other reports [4, 5, 10], a lower initial weight loss increased the risk of not returning the follow-up assessments in our study. However, analyzing the variables in a multivariate model, only weight loss during intervention remained significant in terms of predicting drop-out at the 6-month follow-up, whereas at the 12-month follow-up, neither the baseline weight status nor the initial weight loss was relevant for individual attrition. Moreover, only at the short-term follow-up did drop-out rates differ between treatment groups, with parents in the intervention group seemingly more willing to return the follow-up data. This group took part in a weekend seminar, which stressed the important role of the parents in supporting their child. Since the two treatment groups did not differ in terms of BMI-SDS, this observation is not merely an effect of treatment effectiveness.

Previous research results concerning the socio-demographic data were controversial. We observed no influence from the child’s age, which contradicts the results of two other studies [4, 5]. Consistent with the literature, no effects of gender were observed [4, 5, 7, 8]. In our study, parents’ age influenced the retention rate for the short-term follow-up, an effect that was no longer visible 6 months later. Only at the 12-month follow-up was a higher level of parents’ education associated with a higher retention rate. We are aware of only one study in the field of pediatric weight management that reported similar results, even if only as tendencies [17]. With regard to psychological characteristics, the available evidence is sparse. In line with our observation regarding the differences between completers and non-completers, McCallum et al. [6] indicated that those who dropped out reported a lower quality of life for children. In addition, we found more psychological problems reported by the parents in the drop-out group. This concurs with the findings, with respect to prematurely stopping the intervention [10, 13, 17]. However, the reported differences in psychological characteristics in this study were not independent predictors in the multivariate logistic regression.

Further aspects that differ between the groups were the eating environment, in which a low degree of family meal structure remained a significant predictor for attrition at 12 months after intervention. The influence of the family eating environment may be interpreted as sign of a successful change in family habits – a major goal in pediatric weight loss interventions [32, 33]. In addition, studies from family-oriented intervention approaches suggest that parents play a key role in sustaining the treatment effect at the long-term follow-up [34, 35]. Our results are further supported by the data of other reports that stress parental influence in treatment adherence [10, 17].

Our study is limited in several ways. First, we could not assess any data at the follow-up measurements concerning the reasons for discontinuing the study. Some participants reported that when called by phone, they lost interest and that completing the questionnaires was too time-consuming. This is in line with the data reported by Savoye et al. [8]. and with our previous study [29]. In addition, our analyses were exploratory and we included many variables. We decided to take that path in order to not miss relevant features, and were willing to accept the increased risk of overestimating the clinical significance of the results. Furthermore, we also ran multivariate analyses in order to combat alpha error inflation. When interpreting our results in terms of comparing the pattern of short- and long-term follow-up data, it should be noted that we invested much more effort in gathering long-term data. Especially at the 12-month follow-up, we successfully contacted those parents who had simply forgotten to fill in the questionnaires due to time constraints or other priorities. Thus, we were able to observe differences otherwise obscured by organizational problems reported by the majority of parents [8].

An important strength of our study is that we collected data from a highly diverse and representative sample of participants, rather than only including those families with a higher level of education or income. Moreover, the weight data were based on blind assessment, thus decreasing the risk of underreporting weight status (see self-reported measurements) [36].

## Conclusions

Our study results suggest that the strategies to reduce the attrition rate, which we employed after the first follow-up, were successful, but still could not prevent a final attrition rate of over 33 % for the second follow-up.

We were able to replicate differences between completers and non-completers found in previous studies. However, we observed a different pattern of predictors between the short-term (6 months) and long-term (12 months after intervention) follow-ups. De Niet et al. [10] also found different predictors for attrition at different stages of treatment. Whereas at the 6-month follow-up, mainly aspects associated with the intervention (randomization in the intervention group and initial weight loss) predicted the retention, at the 12-month follow-up, only family aspects such as educational level and family eating environment remained predictive. These differences indicate the risks inherent in only including a short-term follow-up, which might be biased in terms of success rates – especially when drop-out rates are high. Taking into account that in particular, short-term success in weight management does not necessarily imply positive long-term results, intervention studies should include longer follow-up periods and report their results on the basis of intention-to-treat analyses.

## Abbreviations

95 % CI: Confidence interval
BMI: Body mass index
BMI-SDS: Body mass index - standard deviation score
CG: Control group
df: Degrees of freedom
EPOC: Empowering parents of obese children
Exp(B): Odds ratio
F: Statistical test value
HRQoL: Health-related quality of life
IG: Intervention group
OR: Odds ratio
p: Significance value
QoL: Quality of life
RCT: Randomized controlled trial
SES: Socioeconomic status
Wald: Statistical test
WRQoL: Weight-related quality of life
η2: Explained variance
